# Unemployed and Unemployables

**Published:** 1921-01-15

**Authors:** 


### THE PUBLIC WELL-BEING.

### Unemployed and Unemployables.

### AN ANALYSIS OF THE SITUATION.

Nobody can give an exact estimate of the number of unemployed persons in England and Wales at the present time, but in all probability the number is round about a million, and is likely to mount still higher in the coming weeks. Now, unemployment on a large scale, and the extensive under-employment which gradually comes about in a prolonged period of slack trade, is obviously a harmful factor in the national life. Apart from the direct consequences of suffering and hardship which it entails upon thousands of families, its indirect effects are as real, and more far-reaching. It adds mental strain (in anxiety as to the future) to the other cares of millions who are still at work, but who are never sure when it may be their turn to be out of their jobs; it unsettles the minds of people of all classes; it lowers the vitality and resisting-power against disease of a considerable element in the community, and nearly always produces a proportionate rise in the curve of illness and mortality; and it deflects the course of national and local administration from the objects of normal progress, and tends to complicate ordinary measures for the care of public health.

### A Comparison with Former Years.

Having said as much, let us be frank about the present difficulties, and admit that the purely modern impulse to discover a sensation and then boom it has produced a dangerous exaggeration of the actual conditions which prevail. The figures, taking them at the maximum estimate, are, after all, scarcely any higher than they were in 1907 and 1908, both years of unemployment "crisis"; and there is nothing in the existing distress to compare with the conditions that occurred after the Napoleonic wars. War is always followed by a reaction of this kind, varying in length and intensity according to the length and severity of the war, and the really surprising thing is that the reaction from which we are suffering to-day is so relatively mild.

### A Drastic and Painful Remedy.

In some respects it may not prove to be an unmixed evil, if it does not run a too protracted course. It may be regarded, indeed, as a drastic and painful remedy for the wholly false conception of national and individual wealth which followed the signing of the armistice and continued unabated until the late summer of last year. An orgy of spending by all classes; wholesale profiteering by manufacturers and all others, the greater and the less, who had goods to sell; a ca' canny policy by the workers in practically every industry; the vicious circle in which wages were always pursuing prices, only to be overtaken at the next revolution of the wheel; the total disregard by State Departments of the low state of the national finances—these things were significant of the general blindness to the bitter truth that the country was poorer owing to its tremendous four years' struggle than it had been for fifty years, and led inevitably to the crisis which is now upon us. Already there are signs that the slump in trade has had a sobering effect on the community. Prices are coming down; individuals are at last beginning to act with prudence and economy in the ordering of their lives; workers, having saved no money from high wages, are really anxious to work harder in order to live decently; and the Government is cutting down ruthlessly all spending Departments and trying to restore foreign markets for the benefit of British traders. That result is distinctly something to the good.

### Danger of Indiscriminate Relief.

Meanwhile the peculiar difficulties of the present situation arise from two main causes. In the first place the standard of living is higher, the workers have higher expectations, and therefore they entertain quite properly a new point of view with regard to unemployment, and will not accept the tentative and half-hearted remedies of pre-war days. Again, the organisation of the unemployed in large cities into something very like military formations has created a disagreeable problem for local authorities, some of whom—as at Norwich—have been rushed into panicky measures, which have cast further heavy burdens on the tottering ratepayer, and resulted in chaotic and indiscriminate relief a great deal of which has gone to astute persons in no need whatever of help. The bands of genuine unemployed have been joined with alacrity by criminals, hopeless unemployables, revolutionary agitators, and others with an axe to grind or the intention of getting something for nothing. Enormous sums have been collected from the public by unauthorised persons; and, in too many cases, it is to be feared, little enough has gone to those really in need of assistance.

### Unemployed Registers Needed.

Whatever scheme of amelioration is put forward by the Government or the large municipalities, the first desideratum is the preparation of trustworthy and efficient unemployed registers, so that the sheep may be unmistakably separated from the goats. Until and unless that is done half the work of relief will be wasted, and a public eager to help will be chary of contributing to the various relief funds started in different parts of the country.

